# Donor-Derived Cell-Free DNA in Kidney Transplantation: Origins, Present and a Look to the Future

**DOI:** 10.3390/medicina57050482

**Published:** 2021-05-12

**Authors:** Sam Kant, Daniel C. Brennan

**Affiliations:** 1 Division of Nephrology, Department of Medicine, Johns Hopkins University School of Medicine, Baltimore, MD 21287, USA; dbrenna4@jhmi.edu; 2 Comprehensive Transplant Center, Johns Hopkins University School of Medicine, Baltimore, MD 21287, USA

**Keywords:** donor-derived cell-free DNA, kidney transplantation, rejection

## Abstract

Since its first detection in 1948, donor-derived cell-free DNA (dd-cfDNA) has been employed for a myriad of indications in various medical specialties. It has had a far-reaching impact in solid organ transplantation, with the most widespread utilization in kidney transplantation for the surveillance and detection of allograft rejection. The purpose of this review is to track the arc of this revolutionary test—from origins to current use—along with examining challenges and future prospects though the lens of transplant nephrology.

## 1. Introduction

The practice of kidney transplantation has made immense progress in the last two decades, with sustained improvement in allograft longevity and patient outcomes. This has been possible due to a concerted development of immunosuppressive agents and surgical techniques, better understanding of transplant immunology and enhanced surveillance of allograft function. The surveillance of allograft function has traditionally relied on serum creatinine, urinary indices and kidney biopsy. Serum creatinine and urinary indices are not specific for identifying allograft rejection, while the gold standard of kidney biopsy is associated with high cost, sampling errors, risk of bleeding and organ injury [1].

Donor-derived cell-free DNA (dd-cfDNA) is a non-invasive biomarker now being increasingly utilized in discerning allograft rejection. Cell-free DNA was first detected clinically by French investigators in 1948, eventually being tested to ascertain the effect of chemotherapy in various cancers in 1977 [2,3]. Its use has expanded to obstetrics with using fetal cfDNA in maternal blood to detect chromosomal abnormalities and assess Rh incompatibility [4,5].

The utilization of dd-cfDNA entered mainstream solid organ transplantation with its first demonstration in the plasma of kidney and liver transplant recipients in 1998 [6]. This finding was preceded by the theory that dd-cfDNA was present in the recipient plasma while investigating donor microchimerisms post-transplantation [7]. These findings have generated multiple studies that have now confirmed its use in detecting allograft rejection and beyond.

## 2. Current Data and Utility of dd-cfDNA in Kidney Transplantation

Acute cellular rejection (ACR) and antibody-mediated rejection (ABMR) are the two major forms of allograft injury for which dd-cfDNA has been extensively studied. At the time of writing, 22 studies have been performed to demonstrate the utility of dd-cfDNA in kidney transplantation. The diagnosing acute rejection in kidney transplant recipients (DART) study (*n* = 384) has provided the most comprehensive elucidation of the role of dd-cfDNA in detecting rejection, with the following findings [8]:

1. The dd-cfDNA discriminated between biopsy specimens showing any rejection (ACR and ABMR) and controls (no histological rejection).
2. The positive and negative predictive values for active rejection at a cut-off of 1.0% dd-cfDNA were 61% and 84%, respectively.
3. Higher dd-cfDNA levels were detected in cases with biopsy-proven ACR 1B (median 1.2%), whereas biopsies with ACR 1A (median 0.3%), acute tubular necrosis (ATN) and calcineurin inhibitor toxicity had comparatively lower levels of dd-cfDNA.
4. The AUC for discriminating ABMR from samples without ABMR was 0.87 (95% CI, 0.75 to 0.97). The positive and negative predictive values for ABMR at a cut-off of 1.0% dd-cfDNA were 44% and 96%, respectively.
5. Median dd-cfDNA was 2.9% (ABMR), 1.2% (T cell-mediated types ≥ IB) and 0.2% (T cell-mediated type IA) and 0.3% in controls (*p* = 0.05 for T cell-mediated rejection types ≥ IB versus controls).

Further analyses post-DART have shown that dd-cfDNA is more accurate than changes in serum creatinine in detecting rejection [9].

With respect to ACR, dd-cfDNA levels were lower in T cell-mediated rejection (TCMR) Banff 1A with median values of 0.2% in the DART study, with similar values demonstrated in another study [8,10]. This finding has been further validated in two meta-analyses showing that dd-cfDNA could not distinguish milder forms of TCMR from no rejection [11,12]. Although utilizing alternate assays of dd-cfDNA may enhance detection of TCMR [13], it may still lack optimal sensitivity in detecting all cases of TCMR.

The dd-cfDNA level appears to be a better predictor of ABMR in comparison to ACR. Median levels of dd-cfDNA have been noted to be much higher, ranging between 1.4% and 2.9% across three studies [8,13,14]. The composite weighted dd-cfDNA median of 2.89% in patients with ABMR, found in a meta-analysis of five studies, is in line with similar values demonstrated in the DART study. In addition, dd-cfDNA has been shown to be more abundant in patients with donor-specific antibodies (DSAs) and may aid in early recognition of early ABMR [14,15]. In addition, recent data have demonstrated that higher dd-cfDNA levels are associated with *de novo* DSA formation, furthering the hypothesis that the recognition of donor-derived nucleic acids may contribute to the initiation of ABMR. A combination of elevated dd-cfDNA with concurrent DSAs could, therefore, be used to discriminate ABMR from ACR.

It is also recognized that dd-cfDNA levels can be elevated in conditions beyond rejection. This has expanded the utility of this test to identify patients with higher interstitial fibrosis and tubular atrophy (IFTA) [15]; recurrence of lupus, IgA nephropathy and diabetic nephropathy in allograft [16,17,18]; along with discriminating BK virus-associated nephropathy from BK viremia [19]. These indications, however, require further validation in larger datasets along with recalibration of the cut-off values of dd-cfDNA.

## 3. Challenges and What Lies Ahead

As dd-cfDNA is starting to become an integral part of allograft surveillance, pertinent questions and challenges remain:

1. Accurate Prognostication and Trends: There can be significant variability in serial measurements of dd-cfDNA, with a normal variation of up to 61%. There is a pressing need for defining this variation given the risk of false positives, with eventual unnecessary and potentially harmful allograft biopsies and treatment. In addition, the appropriate timing and frequency of dd-cfDNA testing in surveillance needs be determined. The Kidney Allograft Outcomes Registry (KOAR) study (NCT033226076) will look to elucidate these aspects with planned dd-cfDNA testing at various pre-defined intervals along with planned 12-month allograft biopsies.
2. The Multiple Assay and Histology Predicament: The optimal method of measurement of dd-cfDNA still needs to be elucidated. Absolute graft-derived cfDNA has been shown to be more specific for ABMR, while fractionated estimates are better for detecting chronic active ABMR and ACR [20]. Furthermore, the fractionated estimates may be less accurate in periods of physiologic stress leading to the release of excess recipient leukocyte cfDNA, thereby affecting the resultant ratio [21]. More recent research has demonstrated the superiority of absolute dd-cfDNA in discerning the presence of rejection and graft injury in comparison with the fractionated assay [22]. Larger studies comparing these assays are required and need to be examined in settings beyond rejection with appropriate disease controls.

As discussed previously, the correlation of dd-cfDNA with histological changes may not always be optimal, given the disparate differential values for various types and grades of rejection. The INTERCOMEX (NCT04239703) study will evaluate dd-cfDNA testing performed at the time of indication biopsy against Molecular Microscope^®^ Diagnostic System (MMDx)—a definitive molecular assessment of rejection and injury in kidney biopsies.

3. The Therapeutic Angle: The measurement of cfDNA has aided in assessing treatment response in oncology. This use is now being explored in the realm of kidney transplantation, with two studies underway:
  - Comparison of Tacrolimus Extended-Release (Envarsus XR) to Tacrolimus Immediate-Release in HLA Sensitized Kidney Transplant Recipients (NCT04225988): dd-cfDNA will be measured at 6 and 12 months as a secondary outcome measure, along with assessing biopsy-proven rejection and development of *de novo* DSAs within the first post-transplant year.
  - Longitudinal Changes in Donor-Derived Cell-Free DNA With Tocilizumab Treatment for Chronic Antibody-Mediated Rejection (NCT03859388): change in the percentage of dd-cfDNA will be a primary outcome measure in patients undergoing monthly treatment with tocilizumab for chronic ABMR, along with undergoing a follow-up biopsy after 6 months.

## 4. Conclusions

The detection of dd-cfDNA has altered the landscape of surveillance of solid organ allografts. It provides us with an avenue for avoiding risk-laden allograft biopsies at a fraction of the cost. While studies have extensively demonstrated its utility in allograft rejection, the next frontier is refining this test further and validating its use beyond the originally intended identification of rejection.

## Data Availability

No new data were created or analyzed in this study. Data sharing is not applicable to this article.
